# Differential diagnosis of diffuse sclerotic thyroid papillary carcinoma and Hashimoto's thyroiditis using fine‐needle aspiration cytology, BRAF^V600E^, and ultrasound elastography

**DOI:** 10.1002/jcu.23260

**Published:** 2022-07-02

**Authors:** Xian Wang, Feiju Xu, Juan Gao, Enock Adjei Agyekum, Hui Sun, Guoliang Zhang, Xinxin Li, Hong Xiang, Shudong Hu, Xiaoqin Qian

**Affiliations:** ^1^ Department of Ultrasound Affiliated People's Hospital of Jiangsu University Zhenjiang China; ^2^ School of Medicine Jiangsu University Zhenjiang China; ^3^ Department of Pathology Affiliated People's Hospital of Jiangsu University Zhenjiang China; ^4^ Department of General Surgery Affiliated People's Hospital of Jiangsu University Zhenjiang China; ^5^ Department of Otolaryngology head and Neck Surgery Affiliated People's Hospital of Jiangsu University Zhenjiang China; ^6^ Department of Pediatric Affiliated Hospital of Jiangsu University Zhenjiang China; ^7^ Department of Radiology Affiliated Hospital, Jiangnan University Wuxi China

**Keywords:** *BRAF* ^V600E^, diffuse sclerosing variant, elastic strain rate, FNAC, papillary thyroid carcinoma, ultrasound

## Abstract

**Background:**

The diffuse sclerosing variant of papillary thyroid carcinoma (DSV‐PTC) has ultrasound findings that are similar to Hashimoto's thyroiditis (HT), resulting in under‐diagnosis. DSV‐PTC combined with HT is also common, so early and accurate diagnosis of DSV‐PTC using a variety of diagnostic techniques, including FNAC, BRAF^V600E^ mutation detection, and ultrasound elastography, is critical.

**Objective:**

To assess the diagnostic value of fine‐needle aspiration cytology (FNAC) and BRAF^V600E^ detection in combination with ultrasound elastography in the diagnosis of DSV‐PTC.

**Methods:**

We performed a retrospective analysis of 40 patients with pathologically confirmed DSV‐PTC and 43 patients with HT admitted to our hospital's ultrasound department between January 2015 and December 2020. Preoperative FNAC, BRAF^V600E^ mutation detection, and ultrasound elastography imaging were all performed on all patients. For a definitive diagnosis, the results of these tests were compared to postoperative pathological findings. The diagnostic value of FNAC, BRAF^V600E^ mutation detection, ultrasound elasticity imaging, and their combination for DSV‐PTC diagnosis was assessed.

**Results:**

The mean elastic strain rate ratio (E1/E2) of the 40 DSV‐PTC cases was 5.75 ± 2.14, while that of the 43 HT cases was 2.81 ± 1.20. The receiver operating characteristic (ROC) curve was generated using the average value of E2/E1. The area under the ROC curve was 0.910, and the optimal E2/E1 cut‐off value was 4.500. When FNAC, BRAF^V600E^ mutation detection, and ultrasound elasticity imaging detection were combined, the diagnostic sensitivity, specificity, negative predictive value, positive predictive value, and accuracy of DSV‐PTC diagnosis were 92.5%, 95.3%, 93.2%, 94.9%, and 94.0%, respectively, which were significantly higher than the single technique (*p* < 0.05).

**Conclusions:**

The use of FNAC, BRAF^V600E^ mutation detection, and ultrasound elastography in combination is more helpful in establishing an accurate diagnosis of DSV‐PTC than using a single diagnostic technique alone.

## Graphical abstract

The combined application of FNAC, *BRAF* V600E mutation detection, and ultrasound elastography is more helpful in formulating an accurate diagnosis of DSV‐PTC than the use of a single diagnostic technique alone.

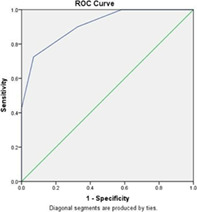

**Abbreviations**
DSV‐PTC: diffuse sclerosing variant of papillary thyroid carcinoma
E1/E2: elastic strain rate ratio of the tissue around the lesion to the elastic strain rate of the lesion
FNAC: fine needle aspiration cytology
HT: Hashimoto's thyroiditis
NPV: negative predictive value
PPV: positive predictive value
PTC: papillary thyroid carcinoma
ROC: receiver operating characteristic

## 1 INTRODUCTION

Papillary thyroid carcinoma (PTC) is the most common type of thyroid cancer, accounting for more than 70% of all thyroid cancers. Although the tumor, node, and metastasis staging system is the most commonly used parameter for determining therapeutic plans, recent research has shown that different histopathologic variants of PTC can have different clinical courses and patient prognoses. PTC sonographic criteria include a taller‐than‐wide shape, an irregular margin, microcalcifications, and marked hypoechogenicity. Sonography's role has expanded to allow for the characterization of PTC variants based on sonographic features. Tall cell and diffuse sclerosing PTC appear to have more aggressive clinical courses and poor prognoses.

Diffuse sclerosing papillary thyroid carcinoma (DSV‐PTC) accounts for 0.7%–6.6% of all papillary thyroid carcinomas.^1^, ^2^ It is more invasive than traditional PTCs, with higher rates of recurrence and metastasis. Hashimoto's thyroiditis (HT) is chronic lymphocytic thyroiditis.^3^, ^4^ Its ultrasound characteristics are similar to those of DSV‐PTC, resulting in a missed or incorrect diagnosis. Cases of DSV‐PTC with HT are also fairly common; thus, early detection of DSV‐PTC is critical. Furthermore, improving the accuracy of preoperative imaging diagnosis is critical in clinical treatment selection.^5^, ^6^

Fine needle aspiration cytology (FNAC) is the preferred method for preoperative assessment of benign and malignant thyroid lesions,^7^, ^8^ but it can produce false‐negative results due to the operator's technical skill, sampling area, and incomplete sampling. Gene identification is currently a research hotspot in the diagnosis of PTC. The BRAF^V600E^ mutation is found in approximately 60% of PTC patients.^9^, ^10^ In the diagnosis of PTC, BRAF^V600E^ has high specificity. However, there are few references in diffuse thyroid disease, particularly in DSV‐PTC diagnosis.

In recent years, ultrasound elastography has emerged as ultrasonic imaging technology. The hardness of thyroid lesions can be estimated by measuring the degree of deformation after compression, and the histological characteristics of thyroid lesions can be determined by revealing the elastic hardness.^11^, ^12^ The strain rate ratio is a newly developed parameter for calculating the hardness of a tissue quantitatively; it avoids the subjectivity of elastic graph grading. The hardness of tissue can be quantified by calculating the ratio of the elastic strain rate of the tissue surrounding the lesion to the elastic strain rate of the lesion (E1/E2), which can be used to differentiate between benign and malignant tissues.

Some researchers are currently using real‐time ultrasound elastography to diagnose thyroid cancer,^12^, ^13^ but there are few reports on the use of conventional ultrasound combined with real‐time ultrasound elastography in the analysis of DSV‐PTC ultrasound imaging features.

In this study, DSV‐PTC was diagnosed using preoperative FNAC, BRAF^V600E^ mutation detection, and ultrasound elastography, and the diagnostic value of combining preoperative FNAC, BRAF^V600E^ mutation detection, and ultrasound elastography were assessed.

## 2 MATERIALS AND METHODS

### 2.1 Subjects

Between January 2015 and December 2020, 121 patients underwent thyroidectomy in our hospital, with all of them undergoing preoperative procedures such as FNAC, BRAF^V600E^ mutation detection, and routine preoperative ultrasound for thyroid calcification. We identified 45 DSV‐PTC cases, 43 HT cases, 20 thyroid adenoma cases, and 13 subacute thyroiditis cases based on postoperative pathological findings. Given the difficulty of establishing a preoperative differential diagnosis between DSV‐PTC and HT, this study included 40 DSV‐PTC cases and 43 HT cases. (1) FNAC indications; (2) surgical treatment; and (3) pathology revealing DSV‐PTC or HT were the inclusion criteria. The exclusion criteria were as follows: (1) FNAC contraindications; (2) other tumors or cancers.

The pathological diagnosis of DSV‐PTC, thyroid lymphocytic infiltration, fibrous hyperplasia, squamous metaplasia, and extensive sand granules were the inclusion criteria for the DSV‐PTC group. The sonograms revealed a diffuse thyroid lesion occupying the majority of all of the unilateral or bilateral lobes, as well as stone‐like calcification and cervical lymph node metastasis. The Ethics Committee at our hospital reviewed and approved this study, and the patients provided written informed consent.

### 2.2 Instruments and methods

The diagnostic instruments used were the GE Logiq E9 (GE Medical Systems, American General) and Hitachi Vision Preirus System (Hitachi Medical Corporation, Tokyo, Japan), both of which were equipped with real‐time ultrasonic elastography technology. The frequency of the probe was set to 5–12 MHz. Transverse and longitudinal scanning of the thyroid or the lesion was performed using two‐dimensional grayscale imaging during a conventional ultrasound. The lesion and cervical lymph nodes were observed and recorded for their location, size, shape, edge, boundary, echo, calcification, color blood flow distribution, and elastic grade.

There are four grades of sand granular calcification in thyroid lesions: 0 indicates no calcification; 1 indicates that calcification points were sparsely distributed throughout the lesion; 2 indicates slightly more calcifications of varying thicknesses, and 3 indicates dense calcification throughout the lesion. The degree of blood flow signal in the thyroid gland was also graded into four categories: 0: no blood flow observed in the lesion; 1: sparse spotty blood flow observed in the lesion; 2: blood flow is more distributed within the lesion, and substantial lesions can be distinguished; and 3: abundant blood flow, almost obscuring the two‐dimensional image of the lesion.

Submaxillary lymph nodes were used as normal controls, and abnormal lymph nodes were found to be more than 5 mm in diameter, disordered in structure, loss of medulla, high cortical echo, sand body calcification, and abundant and disordered blood flow signals. Sand granular calcification in lymph nodes was classified into four grades: grade 0 (no calcification), grade 1 (sparse distribution), grade 2 (a few more calcification points of varying thickness), and grade 3 (dense and scattered in lymph nodes). The degree of blood flow signal in lymph nodes was classified into four levels: 0 (no flow); 1, sparse spot‐like blood flow; 2, rich blood flow distribution without affecting parenchymal echo; and 3, rich blood flow completely filling lymph nodes and covering parenchymal echo.

Following routine ultrasound, real‐time ultrasound elastography was initiated. A sampling frame that included the observed lesion, surrounding thyroid tissue and the sternocleidomastoid muscle in front of the thyroid was chosen. The image was viewed, a relatively stable image frame was chosen, and the regions of interest, A and B, were delineated in the sampling frame; A was the lesion area, and B was the reference area, which was the sternocleidomastoid muscle on the ipsilateral side of the thyroid, where the lesion was located. To calculate the elastic strain rate in the lesion area, the instrument system calculated the average elastic strain variable and the ratio of the two regions of interest.

Patients undergoing FNAC were positioned supine, with a soft pillow on the neck and shoulder, head, back, and anterior neck exposed. Prior to surgery, ultrasound positioning and conventional disinfection were performed. Under the guidance of an ultrasound, a 22G fine needle was used for multi‐point puncture and suction of materials in the area with a high concentration of thyroid calcification and suspicious low‐echo areas. The suspected malignancy was punctured ≥5 times with multiple points of lift and insert, and 4–6 smears were taken and fixed with 95% ethanol. The FNAC procedure for suspicious lymph nodes is the same as described above.

The FNA cell extract was injected into Eppendorf tubes and stored at −80°C. Following the puncture, the patient was instructed to compress and wrap the neck with sterile gauze for 20 min before being observed for 30 min. The patient was instructed to leave the hospital after a color ultrasound confirmed that there was no neck bleeding. According to the manufacturer's instructions, we performed an immunohistochemical analysis of BRAF^V600E^ expression (all markers were purchased from Dako, Dakopatts, Denmark).

### 2.3 Diagnostic criteria for DSV‐PTC

At least one sonographer performed the preoperative ultrasound examination and diagnosis. Any disagreements were settled with the help of a second ultrasound doctor. DSV‐PTC ultrasound images were evaluated for gland (unilateral/bilateral) enlargement and abnormally hypoechoic and diffuse microcalcification (“blizzard‐like” changes) with or without nodular lesions (nodules are often ill‐defined). Lymph node diameter >5 mm, irregular structure, the disappearance of the medulla, increased cortical echogenicity, sand granular calcification, and rich and disordered blood flow signals were all criteria for cervical metastatic lymph nodes. The following histological criteria were used to diagnose DSV‐PTC: (1) diffuse gland enlargement with calcification, (2) extensive squamous metaplasia, (3) dense fibrosis, (4) a large number of sand and gravel bodies, and (5) massive lymphocytic infiltration.

### 2.4 Statistical analyses

SPSS version 20.0 statistical software was used to conduct the statistical analyses (International Business Machines Corp., Armonk, NY, USA). Data from measurements are expressed as mean ± standard deviation. To determine the critical point of differentiation, the pathological findings were used to draw the receiver operating characteristic (ROC) curve. The results of FNAC and BRAF^V600E^ mutation detection were combined with real‐time ultrasound elastography findings and compared to pathological findings. The sensitivity, specificity, positive predictive value (PPV), negative predictive value (NPV), and accuracy were calculated, and the rates were compared using the chi‐square test. A *p*‐value of <0.05 was considered statistically significant

## 3 RESULTS

### 3.1 Comparison of clinical data and ultrasound characteristics between patients with DSV‐PTC and HT

The study included 83 DSV‐PTC and HT patients (age: 46.78 ± 13.18 years; male/female ratio, 35/48). Table 1 shows a comparison of sonographic feature data from the DSV‐PTC and HT groups. The DSV‐PTC group included 15 male and 25 female patients ranging in age from 25 to 60 (41.82 ± 13.85). Twelve had dysphagia, 14 had hoarseness, and 10 had anterior cervical lymph node enlargement (region VI). Based on postoperative pathological findings, all patients underwent thyroidectomy and were diagnosed with DSV‐PTC. Thyroid ultrasonography revealed a full gland shape and increased echogenicity in the 40 DSV‐PTC patients. Microcalcifications were found in the bilateral lobes and isthmus of 28 patients (28/40), the unilateral lobes of 20 patients (20/40), and hypoechoic nodules with blurred borders in 11 patients (11/40), and acoustic abnormalities in one or both cervical lymph nodes in 23 patients (23/40). The HT group included 20 male and 23 female patients ranging in age from 20 to 56 (43.06 ± 13. 53) years. There were 29 cases of diffuse thyroid lobe enlargement, 12 cases of hypothyroidism, and 18 cases of hyperthyroidism.

**TABLE 1:** Comparison of clinical data between patients with DSV‐PTC and HT

Sonographic features	DSV‐PTC (*n* = 40)	HT (*n* = 43)	*p*‐value
Scope of lesions			
Diffuse	18	20	0.890
Localized	22	23	
Calcification			
Sand body	33	8	0.000
Bulky calcification	4	10	
None	3	25	
Cervical lymph node metastasis			
Absent	5	43	0.000
Present	35	0	
Sand granular calcification in LN			
0	3	36	0.000
1	4	4	
2	24	2	
3	9	1	
Intralymph flow			
0	7	15	0.060
1	15	20	
2	14	6	
3	4	2	
Intralymph node echo			
Hypoecho	0	0	0.000
Equal echo	7	33	
On the high side of the echo	30	10	
Complete cystic change	1	0	
Partial cystic changes	2	0	
Thyroid volume			
Enlargement	34	36	
Normal	6	7	
Internal thyroid blood flow			
Increase	15	18	0.103
Normal	20	13	
Reduce	5	12	

Abbreviations: DSV‐PTC, diffuse sclerosing variant of papillary thyroid carcinoma; HT, Hashimoto's thyroiditis.

### 3.2 Comparison of ultrasonic characteristics between DSV‐PTC and HT cervical metastatic lymph nodes was analyzed

**FIGURE 1:**
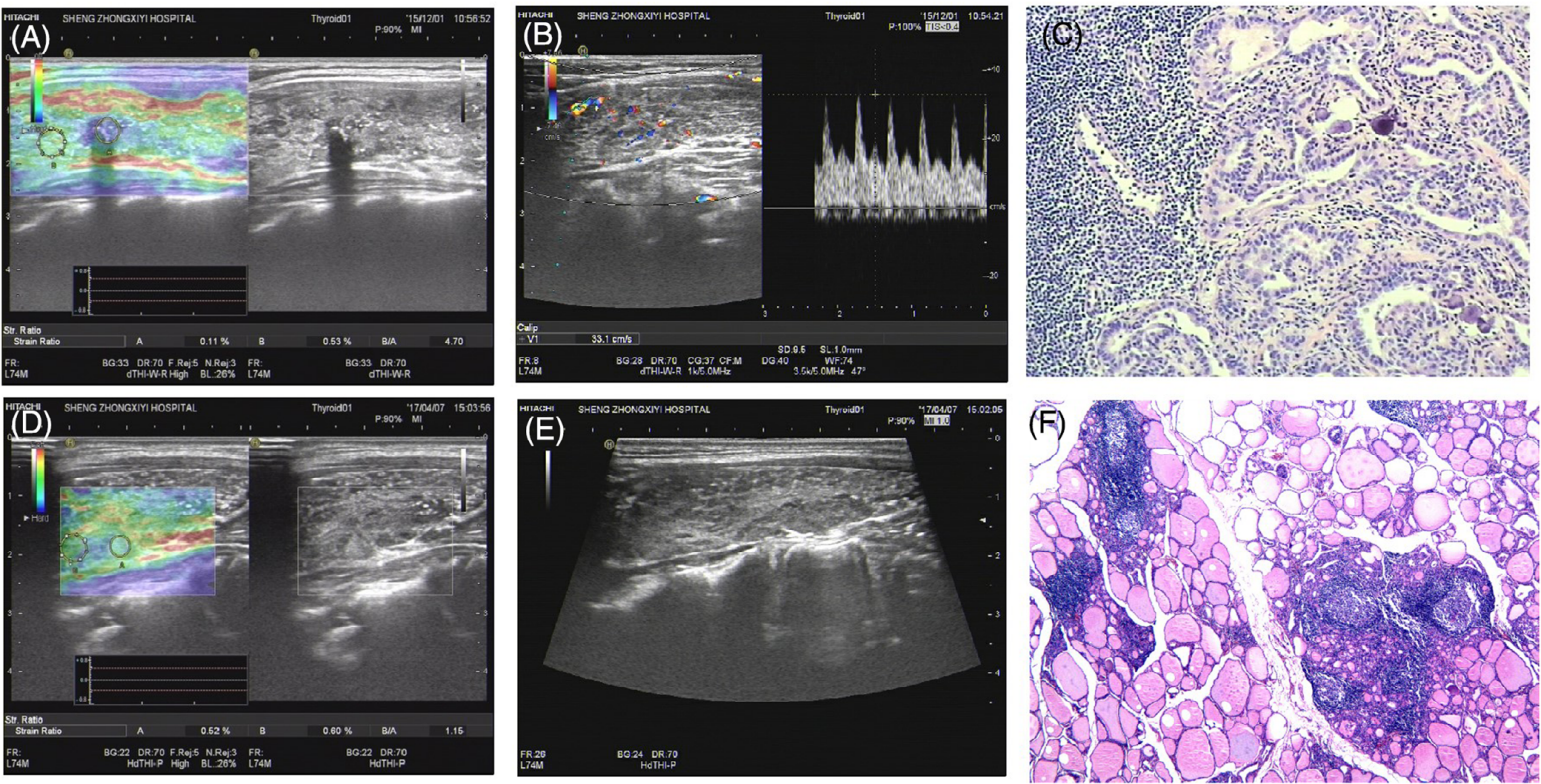
(A–C). Pathology results showing DSV‐PTC. In the lower right portion, there is a disordered region with a flake‐like structure (size 0.6 cm × 0.7 cm); the central part is a low‐echo region, and the diffuse distribution of strong echo signals can be observed inside. The following can be observed: no comet tail; blood flow level in the nodules; marginal elasticity, grade 4; strain rate, 0.11%; and E2/E1, 4.70.；Pathology: DSV‐PTC (HE, ×40). (D–E). Pathology results showing HT. The thyroid gland has a normal size and shape, smooth surface, complete capsule, internal echo thickening and heterogeneity, visible network structure, and normal internal blood flow distribution. The elasticity score is grade 2, the strain rate is 0.52%, and E2/E1 is 1.15. Pathology: HT (HE, ×40). DSV‐PTC, diffuse sclerosing variant of papillary thyroid carcinoma; HT, Hashimoto's thyroiditis

Thirty‐five of the 40 DSV‐PTC cervical lymph node metastases measured 12–21 mm in diameter. DSV‐PTC cervical lymph node metastases were oval or round in shape, with a rough capsule surface. The echo of affected lymph nodes is similar to that of the thyroid, with internal heterogeneity and patchy foci. Grainy calcification was found in lymph nodes, which were scattered or clustered (Figure 1). Cystic changes in lymph nodes were found in four cases. There were 31 HT cases with enlarged lymph nodes in the anterior cervical VI region. The enlarged lymph nodes had the following ultrasonographic characteristics: reduced aspect ratio, full shape, decreased parenchymal echo and disappeared medulla and lymphatic hilum echo. 81.4% of patients had symmetrically distributed bilateral lymph nodes in area VI (See Table 1).

### 3.3 Analysis of the diagnosis of DSV‐PTC and HT using the elastic strain rate ratio method

**FIGURE 2:**
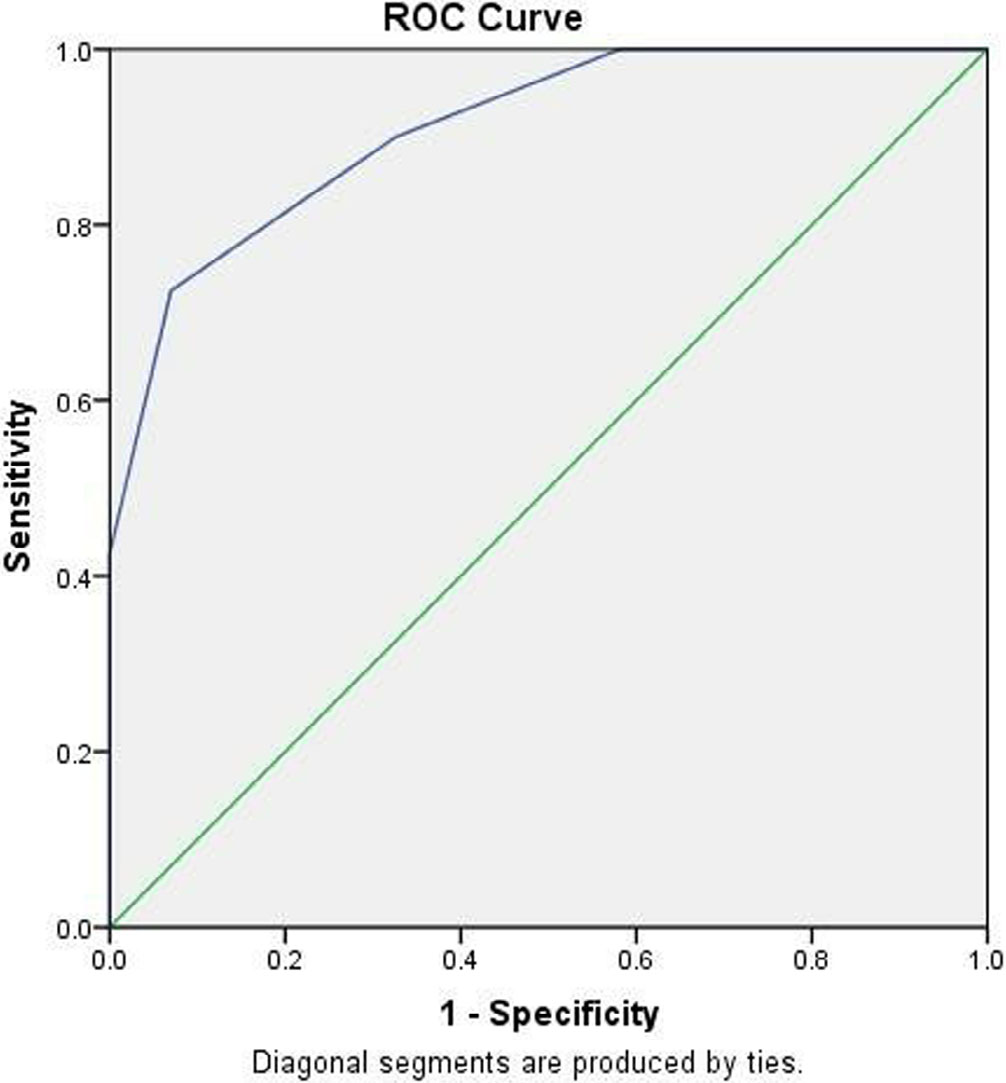
ROC curve of the diagnosis of DSV‐PTC by the elastic strain rate ratio. DSV‐PTC, diffuse sclerosing variant papillary thyroid carcinoma; ROC, receiver operating characteristic

In this study, 40 cases of DSV‐PTC and 43 cases of HT were pathologically confirmed. According to the pathological examination results, the mean elastic strain rate ratio of the 40 DSV‐PTC cases was 5.75 ± 2.14, while that of the 43 HT cases was 2.81 ± 1.20 (Figure 1). Notably, the mean elastic strain rate ratio of DSV‐PTC was significantly higher than that of HT (*p* = 0.000). The ROC curve was drawn using the average value of E2/E1. The area under the ROC curve was 0.910, and the optimal critical point of E2/E1 was 4.500 (Figure 2). As shown in Table 2, the mean elastic strain rate ratio in the preoperative diagnosis of DSV‐PTC had a sensitivity of 70.0%, specificity of 69.8%, NPV of 71.4%, PPV of 68.3%, and accuracy of 69.9% when compared to postoperative pathological findings.

**TABLE 2:** The area under the curve diagnosis of DSV‐PTC by elastic strain rate ratio method

Test result variable:strain ratio				
Area	Standard error	Asymptotic Sig. b	Asymptotic 95% confidence interval	
			Lower limit	Upper limit
0.910	0.030	0.000	0.850	0.969

### 3.4 Results of FNAC, *BRAF* V600E mutation, and combined application of the two methods compared with postoperative pathological findings

All 40 patients with DSV‐PTC confirmed by operation and pathology underwent FNAC in the suspected thyroid gland region guided by ultrasonography; the pathological satisfaction rate of cell volume for a specimen smear was 100%. Cytological pathology revealed that 31 patients had PTC, seven had unspecified atypical lesions, and two had lymphocytic thyroiditis.

Surgical pathology confirmed 40 cases of DSV‐PTC with lymph node metastasis in the central region of the neck. The FNAC results of the 40 cases revealed 32 cases of metastatic lymph nodes and eight cases of the non‐metastatic lymph node. In comparison to postoperative pathological findings, the sensitivity, specificity, NPV, PPV, and accuracy of the preoperative diagnosis of PTC using FNAC results were 77.5%, 88.3%, 80.8%, 86.1%, and 83.1%, respectively. The BRAF^V600E^ mutation was found in four of the 40 patients diagnosed with DSV‐PTC postoperatively but was not found in 43 patients with postoperative pathological diagnosis of HT.

In comparison to postoperative pathological findings, the sensitivity, specificity, NPV, PPV, and accuracy of the preoperative diagnosis of PTC using BRAF^V600E^ mutation detection were 10%, 100%, 54.4%, 100%, and 56.6%, respectively.

The sensitivity, specificity, NPV, PPV, and accuracy of the preoperative diagnosis of DSV‐PTC using FNAC and BRAF^V600E^ mutation results were 80.0%, 86.0%, 80.9%, 78.0%, and 79.2%, respectively, when compared to the postoperative pathological findings.

### 3.5 Combined FNAC, *BRAF* V600E mutation detection, and elastic strain rate ratio method and postoperative pathology comparison

Table 3 compares the sensitivity, specificity, PPV, NPV, and accuracy of FNAC and BRAF^V600E^ mutation detection in DSV‐PTC diagnosis using the elastic strain rate ratio method. The results showed that combining the three methods significantly improved the sensitivity, specificity, NPV, PPV, and accuracy of the diagnosis. When the three methods were combined and compared to FNAC, BRAF^V600E^ mutation detection, and the elastic strain rate method, the differences were significant (*p* = 0.000)

**TABLE 3:** Combined FNAC, *BRAF* V600E mutation, elastic strain rate ratio method diagnosis of DSV‐PTC

	Sensitivity	Specificity	NPV	PPV	Accuracy
FNAC results	77.5%	88.3%	80.8%	86.1%	83.1
*BRAF* V600E mutation	10%	100%	54.4%	100%	56.6%
Elastic strain rate ratio method	70.0%	69.8%	71.4%	68.3%	69.9%
FNAC + *BRAF* V600E mutation	80.0%	86.0%	80.9%	78.0%	79.2%
FNAC + *BRAF* V600E mutation + elastic strain rate ratio method	92.5%	95.3%	93.2%	94.9%	94.0%
*p*	0.000	0.000	0.000	0.000	0.000

## 4 DISCUSSION

DSV‐PTC is a subtype of papillary thyroid carcinoma that accounts for about 1.8% of all cases.^14^, ^15^, ^16^ In contrast to the majority of cases, which have good differentiation, slow growth, and a good prognosis, DSV‐PTC grows quickly and aggressively, is more prone to lymph node and distant metastasis, and has a poor prognosis.^17^, ^18^ Patients with DSV‐PTC usually require a total thyroidectomy and neck dissection, as well as radioactive iodine (I‐131) treatment after surgery. As a result, preoperative imaging diagnosis of DSV‐PTC is extremely important clinically.

In terms of histological characteristics, the ultrasonographic findings of DSV‐PTC differ significantly from those of typical thyroid carcinoma. DSV‐PTC has been shown in studies to cause unilateral or bilateral thyroid involvement (including the isthmus), and ultrasonic characterization includes diffuse enlargement of the thyroid gland and a heterogeneous parenchymal echo pattern, with no non‐substantial mass.^19^, ^20^ Previous research has revealed that the majority of cases are easily misdiagnosed as HT. In our study, 88% of DSV‐PTC patients were misdiagnosed with HT, with three FNAC cases classified as atypical cell lesions of unknown significance and three cases complicated by HT. As a result, distinguishing DSV‐PTC from HT is critical. In this study, the majority of DSV‐PTC involve the visible diffuse distribution of microcalcifications, with only a few microcalcifications having limited distribution. The microcalcification visible on the ultrasound image represents its histology and is a common feature of PTC. DSV‐PTC, on the other hand, is distinguished by a high number of extensive and diffusion sand bodies, phosphorous metaplasia, interstitial fibrosis, and lymphocyte infiltration. As a result, the sand‐like calcification of DSV‐PTC is more extensive and dense. In this study, 32 cases covered the entire affected gland, with a diameter of 1–3 mm, and 5 cases were clustered in clusters. However, all 43 HT cases were characterized by diffuse enlargement of the thyroid gland, and the internal diffuse echo was weakened, and streak‐like and point‐like strong echo could be seen, which was primarily manifested as “network,” which was also one of the distinguishing features of DSV‐PTC and HT.

In this study, 31 HT patients had lymph nodes in neck region VI with decreased aspect ratio, full shape, decreased parenchymal echo, and most medullolymphatic hilum echoes disappeared. This is due to HT‐induced lymphocyte infiltration in the thyroid, the presence of thyroglobulin antibodies, and thyroid peroxidase antibodies in the blood stimulating the lymph system surrounding the thyroid, resulting in excessive hyperplasia of thyroid draining lymph nodes. Under the microscope, lymph nodes in area VI of the neck of HT showed diffuse infiltration of lymphocytes, increased lymphatic nodule in the cortical layer, and increased lymphocytes in the germinal center and medullary cord, which was consistent with the change in medullolymphatic hilum echo loss and homogeneous hypoechoic on an ultrasound image. Blood flow was detected in the metastatic lymph nodes of 35 DSV‐PTC patients with lymph node metastasis in neck region VI, distributed in the center or marginal region of lymph nodes, blood flow in 29 patients with grade 1–2, and blood flow in 4 patients filled the entire lymph node in the shape of “blood cells.” A high degree of malignant invasion of cancer tissue and small blood vessels in the lymph nodes are widely associated expansion, at the same time there are 34 cases of neck metastasis lymph nodes with sand granular calcification, and tiny calcify point sizes, scattered or gather into a cluster distribution within the lymph nodes, this is DSV‐PTC characteristics of the metastatic lymph nodes, can be used for distinguishing DSV‐PTC and HT is one of the key points.

Ultrasound‐guided FNAC is the preferred method for benign and malignant evaluation of thyroid nodules, as recommended by authoritative guidelines such as the National Comprehensive Cancer Network and the American Thyroid Association, as it is a minimally invasive and efficient diagnostic procedure. For ultrasound‐detected thyroid nodules, FNAC can effectively reduce diagnostic hand surgery while increasing the proportion of malignant tumor surgery.^21^ The main ultrasonic manifestation in patients with DSV‐PTC is diffuse microcalcification, and the background is mostly heterogeneous hypoechoic, often lacking typical tumor nodules.^11^ Previous literature reports ultrasound findings of diffuse microcalcification resembling sand granules in pathological key acoustic areas and corresponding pathologic lymphocytic infiltration. As a result, in this study, the puncture sites of PTC patients were all calcification intensive areas, and the samples showed more sand grains and calcification, DSV‐PTC should be considered first when ultrasonographic manifestation of thyroid reveals diffuse microcalcification. However, FNAC for DSV‐PTC frequently leads to a misdiagnosis of HT, particularly in patients with a positive antithyroglobulin antibody test.^6^, ^7^ FNAC puncture in the central area of the neck revealed metastasis, which was confirmed by surgery. As a result, we believe that taking extensive samples of the thyroid microcalcification area and performing FNAC on suspicious lymph nodes in the neck can improve DSV‐PTC preoperative diagnostic accuracy. In this study, the sensitivity, specificity, NPV, PPV, and accuracy of the preoperative diagnosis of DSV‐PTC using FNAC results were 77.5%, 88.3%, 80.8%, 86.1%, and 83.1%, respectively. About 76% of DSV‐PTC patients developed lymphocytic thyroiditis, and 28 patients were diagnosed with metastatic thyroid carcinoma supported by FNAC in suspected cervical lymph nodes. To improve preoperative diagnostic accuracy, extensive samples of the thyroid microcalcification area and FNAC on suspicious lymph nodes in the neck are recommended.

The BRAF^V600E^ mutation is linked to the occurrence of PTC. The BRAF^V600E^ mutation is found in 29%–83% of papillary carcinomas, but not in HT lesions.^9^, ^18^ However, in this study, 36 patients without the BRAF^V600E^ mutation were confirmed to have DSV‐PTC through preoperative pathology but were not detected in any of the 43 cases with HT lesions, indicating that the BRAF^V600E^ mutation is an influential factor for DSV‐PTC rather than HT. Although the BRAF^V600E^ mutation has 100% specificity in the diagnosis of DSV‐PTC, its low mutation rate leads to lower sensitivity and accuracy than FNAC; thus, it does not meet the criteria for a clinical diagnosis of DSV‐PTC. To differentiate DSV‐PTC, the FNAC and BRAF^V600E^ mutation phases were combined. However, the sensitivity, specificity, NPV, PPV, and accuracy of the preoperative diagnosis of DSV‐PTC using FNAC and BRAF^V600E^ mutation results were 80.0%, 86.0%, 80.9%, 78.0%, and 79.2%, respectively, with the sensitivity and specificity of DSV‐PTC not significantly improved. Ultrasound elastography is a relatively new technique for imaging the mechanical properties of tissues.^20^, ^22^ Extensive interstitial fibrosis, lymphocytic infiltration, squamous metaplasia in internal tissues, and a large number of sand granules in DSV‐PTC result in hardness that is clearly greater than the surrounding tissue. There are differences in the degree of lymphocyte phagocytosis and infiltration or replacement by fibrous tissue when DSV‐PTC and HT are in different developmental stages and functional states, so the elastic strain rate ratio will be different. The elastic strain rate ratio method determines the nature of the lesion by measuring the difference in pliability or hardness between normal tissue in the same layer around the lesion and the lesion area. It is a semi‐quantitative parameter that can reflect the relative hardness of the tumor and the surrounding normal tissue.^22^, ^23^ In this study, the mean value of E2/E1 was lower for DSV‐PTC than for HT, with a significant difference (*p* = 0.000), indicating that the elastic strain rate ratio could be used as a diagnostic tool to distinguish between different types of lesions. According to the ROC curve, the ultrasound elasticity strain rate ratio, as a relatively objective semi‐quantitative index, has high accuracy in the diagnosis of DSV‐PTC. DSV‐PTC and conventional ultrasound had no significant differences in sensitivity, specificity, PPV, or NPV (*p* > 0.05). However, several factors must be considered, including the size and location of lesions, necrosis, liquefaction, capsule, calcification, and depth of the lesion, namely, normal area and outline when measuring the size of the area of normal tissue will affect E2/E1.

The BRAF^V600E^ mutation was not detected in some nodules with atypical pathological changes and no malignant diagnosis made with FNAC, so the mean value of E2/E1 was calculated. When compared to FNAC, BRAF^V600E^ mutation detection, and ultrasound elastography strain rate alone, the combination of the three tests can improve the detection rate (sensitivity) of DSV‐PTC. In this study, the sensitivity of diagnosis was increased from 70.7%, 77.5%, and 10% to 92.5%, respectively. Furthermore, patients with a positive FNAC result, a high ultrasound elastography strain rate, and a negative BRAF^V600E^ mutation test had a postoperative pathological confirmation accuracy of 98%. As a result, combining FNAC, BRAF^V600E^ mutation, and ultrasound elasticity in the differential diagnosis of DSV‐PTC improves diagnostic sensitivity and specificity significantly, allowing some DSV‐PTC patients who cannot be clearly diagnosed by US‐FNAC to obtain a scientific and accurate diagnosis before surgery. It provides a reliable imaging and molecular basis for patient follow‐up treatment, as well as the scientific foundation for the formulation of patient follow‐up plans and treatment plans. This study has some limitations. First, this was a retrospective study, and there were some selection biases that may have influenced the results. Second, the sample size of this study is small, and it is expected that future clinical studies with a larger sample size will provide more data for the diagnosis of DSV‐PTC. Third, DSV‐PTC lacks the BRAF^V600E^ mutation, which may have an effect on the research results. In conclusion, combining and analyzing FNAC, BRAF^V600E^ mutation detection, and ultrasonic elastography strain rate ratio improves DSV‐PTC diagnosis over conventional ultrasound alone. The use of FNAC, BRAF^V600E^ mutation detection, and ultrasound elastography together can improve preoperative prediction of DSV‐PTC and assist clinicians in making a more accurate diagnosis.

## AUTHOR CONTRIBUTIONS

Xian Wang, Feiju Xu, Juan Gao contributed equally to this study. Xian Wang, Feiju Xu contributed to the conception and design of the study. Juan Gao, Xiangshu Bo, Hui Sun organized the database. Guoliang Zhang, Xinxin Li, Hong Xiang performed the statistical analysis. Xian Wang and Enock Adjei Agyekum wrote the first draft of the manuscript. Shudong Hu and Xiaoqin Qian wrote sections of the manuscript. All authors contributed to the article and approved the submitted version.

## FUNDING INFORMATION

The authors disclose the receipt of financial support for the research, authorship, and/or publication of this article from the Zhenjiang Commission of Science and Technology (Project No. SH2020046), Medical scientific research project approved by Jiangsu Provincial Health Commission (Z2021071) and Clinical Medicine Science.

## CONFLICT OF INTEREST

The authors declare that the research was conducted in the absence of any commercial or financial relationships that could be construed as a potential conflict of interest.

## Data Availability

The raw data supporting the conclusions of this article will be made available by the authors, without undue reservation.

## References

[1] Sung HK , Jong LR , Gyungyup G , et al. Differences in the recurrence and survival of patients with symptomatic and asymptomatic papillary thyroid carcinoma: an observational study of 11,265 person‐years of follow‐up. Thyroid. 2016;26:1472‐1479. doi:10.1089/thy.2016.0238 27457917

[2] Tuttle RM , Haugen B , Perrier ND . Updated American Joint Committee on cancer/tumor‐node‐metastasis staging system for differentiated and anaplastic thyroid cancer (eighth edition): what changed and why? Thyroid. 2017;27:751‐756. doi:10.1089/thy.2017.0102 28463585 PMC5467103

[3] Sapuppo G , Tavarelli M , Belfiore A , Vigneri R , Pellegriti G . Time to separate persistent from recurrent differentiated thyroid cancer: different conditions with different outcomes. J Clin Endocrinol Metabol. 2019;104:258‐265. doi:10.1210/jc.2018-01383 30165559

[4] Haugen BR , Alexander EK , Bible KC , et al. 2015 American Thyroid Association Management Guidelines for adult patients with thyroid nodules and differentiated thyroid cancer: the American Thyroid Association guidelines task force on thyroid nodules and differentiated thyroid cancer. Thyroid. 2016;26:1‐133. doi:10.1089/thy.2015.0020 26462967 PMC4739132

[5] Akaishi J , Sugino K , Kameyama K , et al. Clinicopathologic features and outcomes in patients with diffuse Sclerosing variant of papillary thyroid carcinoma. World J Surg. 2015;39:1728‐1735. doi:10.1007/s00268-015-3021-9 25743484

[6] Chereau N , Giudicelli X , Pattou F , et al. Diffuse Sclerosing variant of papillary thyroid carcinoma is associated with aggressive histopathological features and a poor outcome: results of a large multicentric study. J Clin Endocrinol Metabol. 2016;101:4603‐4610. doi:10.1210/jc.2016-2341 27626975

[7] Zhu Y , Song Y , Xu G , Fan ZH , Ren WH . Causes of misdiagnoses by thyroid fine‐needle aspiration cytology (FNAC): our experience and a systematic review. Diagn Pathol. 2020;15:1. doi:10.1186/s13000-019-0924-z 31900180 PMC6942345

[8] Mayooran N , Waters PS , Kaim Khani TY , Kerin MJ , Quill D . FNAC and frozen section correlations with definitive histology in thyroid diseases. Eur Arch Otorhinolaryngol. 2016;273:2181‐2184. doi:10.1007/s00405-015-3742-2 26242254

[9] Li DD , Zhang YF , Xu HX , Zhang XP . The role of BRAF in the pathogenesis of thyroid carcinoma. Front Biosci. 2015;20:1068‐1078. doi:10.2741/4359 25961545

[10] Lin Z , Yan C , Song Y , et al. The features of contrast enhanced ultrasound and BRAF V600E in papillary thyroid carcinoma. J Thorac Dis. 2019;11:5071‐5078. doi:10.21037/jtd.2019.11.78 32030223 PMC6988045

[11] Kwak JY , Kim EK , Hong SW , et al. Diffuse sclerosing variant of papillary carcinoma of the thyroid: ultrasound features with histopathological correlation. Clin Radiol. 2007;62:382‐386. doi:10.1016/j.crad.2006.11.015 17331834

[12] Peng Q , Niu C , Zhang M , Peng Q , Chen S . Sonographic characteristics of papillary thyroid carcinoma with coexistent Hashimoto's thyroiditis: conventional ultrasound, acoustic radiation force impulse imaging and contrast‐enhanced ultrasound. Ultrasound Med Biol. 2019;45:471‐480. doi:10.1016/j.ultrasmedbio.2018.10.020 30528690

[13] Xi X , Gao L , Wu Q , et al. Differentiation of thyroid nodules difficult to diagnose with contrast‐enhanced ultrasonography and real‐time elastography. Front Oncol. 2020;10:112. doi:10.3389/fonc.2020.00112 32175270 PMC7056834

[14] Joung JY , Kim TH , Jeong DJ , et al. Diffuse sclerosing variant of papillary thyroid carcinoma: major genetic alterations and prognostic implications. Histopathology. 2016;69:45‐53. doi:10.1111/his.12902 26568156

[15] Kim SK , Park I , Woo J , et al. Follicular and diffuse sclerosing variant papillary thyroid carcinomas as independent predictive factors of loco‐regional recurrence: a comparison study using propensity score matching. Thyroid. 2016;26:1077‐1084. doi:10.1089/thy.2016.0113 27324748

[16] Vuong HG , Kondo T , Pham TQ , et al. Prognostic significance of diffuse sclerosing variant papillary thyroid carcinoma: a systematic review and meta‐analysis. Eur J Endocrinol. 2017;176:433‐441. doi:10.1530/EJE-16-0863 28183787

[17] Wang D , Du L , Sun J , et al. Evaluation of thyroid nodules with coexistent Hashimoto's thyroiditis according to various ultrasound‐based risk stratification systems:a retrospective research. Eur J Radiol. 2020;131:109059. doi:10.1016/j.ejrad.2020.109059 32739109

[18] Zhang Q , Liu B , Ren W , et al. Association between BRAF V600E mutation and ultrasound features in papillary thyroid carcinoma patients with and without Hashimoto's thyroiditis. Sci Rep. 2017;7:4899. doi:10.1038/s41598-017-05153-y 28687736 PMC5501791

[19] Lee JY , Shin JH , Han B , et al. Diffuse sclerosing variant of papillary carcinoma of the thyroid: imaging and cytologic findings. Thyroid. 2007;17:567. doi:10.1089/thy.2006.0321 17614778

[20] Pang T , Huang L , Deng Y , et al. Logistic regression analysis of conventional ultrasonography, strain elastosonography, and contrast‐enhanced ultrasound characteristics for the differentiation of benign and malignant thyroid nodules. PLos One. 2017;12:e188987. doi:10.1371/journal.pone.0188987 PMC5724846 29228030

[21] Poller DN , Johnson SJ , Bongiovanni M . Measures to reduce diagnostic error and improve clinical decision making in thyroid FNA aspiration cytology: a proposed framework. Cancer Cytopathol. 2020;128:917‐927. doi:10.1002/cncy.22309 32543764

[22] Cantisani VD , Andrea V , Mancuso E , et al. Prospective evaluation in 123 patients of strain ratio as provided by quantitative elastosonography and multiparametric ultrasound evaluation (ultrasound score) for the characterisation of thyroid nodules. Radiol Med. 2013;118:1011‐1021. doi:10.1007/s11547-013-0950-y 23807669

[23] Liu H , Zhu Y , Jiao J , Yuan J , Pu TN , Yong Q . ShearWave™ elastography for evaluation of the elasticity of Hashimoto's thyroiditis. Clin Hemorheol Microcirc. 2018;1‐8:9‐16. doi:10.3233/CH-170347 29660914

